# Physiological Correlation between Hypothalamic–Pituitary–Adrenal Axis, Leptin, UCP1 and Lipid Panel in Mares during Late Pregnancy and Early Postpartum Period

**DOI:** 10.3390/ani11072051

**Published:** 2021-07-09

**Authors:** Francesca Arfuso, Claudia Giannetto, Marilena Bazzano, Anna Assenza, Giuseppe Piccione

**Affiliations:** 1 Department of Veterinary Sciences, University of Messina, Polo Universitario dell’Annunziata, 98168 Messina, Italy; farfuso@unime.it (F.A.); anna.assenza@unime.it (A.A.); gpiccione@unime.it (G.P.); 2 School of Biosciences and Veterinary Medicine, University of Camerino, Via Andrea D’Accorso, 16, 62032 Macerata, Italy; marilena.bazzano@unicam.it

**Keywords:** leptin, mares, adrenocorticotrophic hormone, lipids, cortisol, peripartum period

## Abstract

**Simple Summary:**

Several features of periparturient mares’ physiology have been investigated; however, the possible linkage between HPA axis, leptin, and lipid metabolism in periparturient mares has received little attention. Therefore, the present study aimed to investigate the dynamic changes of adrenocorticotrophic hormone, cortisol, leptin, UCP1, lipids and liproteins levels in mares during late pregnancy and the postpartum period. The findings obtained in the current survey showed a dynamic modulation of studied parameters, namely the response to hormonal and metabolic adaptations, occurring during the peripartum period in mares. This study suggests an interaction of the HPA axis with lipid metabolism in mares, probably to deal with the metabolic load occurring during pregnancy and with lactation energy supplies.

**Abstract:**

This study aimed to investigate the dynamic change of adrenocorticotrophic hormone (ACTH), cortisol, leptin, mitochondrial uncoupling protein 1 (UCP1), lipids and lipoproteins in mares during late pregnancy and the postpartum period. A total of 20 mares (10 pregnant mares, monitored from 14 ± 2 days before expected foaling until 14 days after foaling, Group A; 10 non-pregnant and non-lactating mares, Group B) were enrolled in the study. Body Condition Score (BCS) and body weight (BW) values were recorded from each animal. In Group A, blood samples were collected on days 14 ± 2 and 7 ± 2 before foaling (T_−14_; T_−7_), and on days 7 and 14 after foaling (T_+7_; T_+14_). From mares of Group B, blood samples were collected at the beginning of the study. The levels of ACTH, cortisol, leptin, UCP1, non-esterified fatty acids (NEFAs), total cholesterol, high-density lipoproteins (HDLs), low-density lipoproteins (LDLs), triglycerides and very-low-density lipoproteins (VLDLs) were investigated. While BCS showed no statistical change throughout the monitoring period (*p* > 0.05), all the other studied parameters displayed statistically significant variations in Group A over the peripartum period (*p* < 0.0001). A significant effect of pregnancy was found on all studied parameters (*p* < 0.001). The ACTH and cortisol levels measured in mares belonged to Group A showed a significant positive correlation with the values of leptin, LDLs, triglycerides and VLDLs, whereas they were negatively correlated with the serum UCP1 and NEFAs values. Together, the findings gathered in this study highlight a dynamic change of serum leptin, UCP1 and lipid parameters in peripartum mares and suggest an interaction of the HPA axis with lipid metabolism and mobilization in mares during the peripartum period in order to deal with metabolic and energy demand and maintain energy homeostasis.

## 1. Introduction

The Peripartum period is a challenging life interval due to metabolic and energetic demands that dams have to deal with. All these events represent important stressors, which elicit metabolic reaction cascades as a part of the adaptation mechanisms of the organism. Periparturient mares are physiologically unstable and may develop hyperlipidemia, endometritis, subclinical hepatopathy, and reproductive disorders caused by malnutrition [1,2]. It is well known that following stress, a number of hormones including catecholamines, glucocorticoids, growth hormone, and thyroxine have been secreted leading to mobilization of energy sources in order to ensure the organism adaptation to its new circumstance. From the physiological point of view, changes in the endocrine system and metabolism have been described in mares around the parturition. In response to an outside stimulus, signals originating in the brain lead to hormone production that is orchestrated by positive or negative feedback loops that increase or decrease production, respectively. Studies investigating the hormones and substances involved in the stress response of animals clearly showed that the corticotropin-releasing hormone (CRH) is the main responsible for stress response. CRH is produced by the hypothalamus, and it stimulates the production of adrenocorticotrophic hormone (ACTH) by the pituitary gland, which, acting on the adrenal glands, induces the release of hormone cortisol [3,4]. Corticotropin-releasing hormone concentration cannot be quantified in the bloodstream; however, other compounds, including cortisol and leptin, can provide evidence of hypothalamus activity.

Leptin is a hormone present in adipocytes that communicates the nutritional status of the body to the central nervous system acting directly on the function of the hypothalamus, increasing metabolic rate and decreasing food intake [5,6,7,8,9,10,11]. As a matter of fact, leptin is associated with the regulation of body weight and the normal maintenance of bodily functions or homeostasis. It has been hypothesized that leptin may be an important marker in metabolic syndrome. Moreover, there is evidence that among the several peripheral metabolic markers, leptin is also altered by stressful events [5,6,7,8], including the peripartum period [9].

The relationship between the serum leptin levels and the concentration of ACTH and cortisol has been found in patients affected by metabolic syndrome [3]. In addition to a decrease in food intake, higher leptin values lead to an increase in energy expenditure through brown adipose tissue stimulation. Thus, leptin leads to lipolysis and fatty acid oxidation by influencing the expression of the mitochondrial uncoupling protein 1 (UCP1) [12]. Mitochondrial uncoupling protein 1 is enrolled in the pathways of metabolism regulation and energy balance, and its activity is down-regulated by purine nucleotides and up-regulated by fatty acids [13]. Several features of periparturient mare’s physiology have been investigated [9,14,15,16], and, though the influence of leptin concentration on endocrine responses in mares and geldings have been documented [17], the possible linkage between HPA axis, leptin and lipid metabolism in periparturient mares has received little attention.

Therefore, the present study aims to investigate the dynamic changes in adrenocorticotrophic hormone, cortisol, leptin, UCP1, lipid and liprotein levels in mares during late pregnancy and the postpartum period. Moreover, the possible correlation between HAP axis and lipid metabolism and mobilization was assessed in periparturient mares in response to the dynamic physiological adaptation processes characterizing the transition from prepartum to the postpartum period.

## 2. Materials and Methods

### 2.1. Animals and Experimental Design

Treatments, housing, and animal care were carried out in accordance with the standards recommended by the European Directive 2010/63/EU for animal experiments. Twenty clinically healthy mares (8 Italian Saddle, 6 Thoroughbred, 6 Standardbred), aged between 9 and 11 years, were enrolled in the present study. Animals from the same breeding center were housed in individual straw-bedded boxes (4.0 × 4.0 m), and they were moved to paddocks from 10 a.m. to 4 p.m. daily under natural spring photoperiod (sunrise 5:15 a.m. and sunset 7:00 p.m.), in Sicily (latitude 37.46 N; longitude 14.93 E). Ten out of 20 mares enrolled in the study were pregnant mares, monitored from 14 ± 2 days before expected foaling until 14 days after foaling (Group A), whereas the other 10 horses were non-pregnant and non-lactating mares (Group B). All the pregnant mares were delivered in spring (between March and mid-May with a mean gestation length of 340 ± 8 days). The mares were subjected to clinical examinations over three consecutive days following parturition; ultrasound exams were weekly performed on each mare to monitor the uterine involution and ovarian activity. Any clinical signs of disease were observed in animals enrolled in the study throughout the study. Group A delivered healthy, viable full-term foals without assistance. They passed a normal and intact placenta spontaneously within two hours and achieved the complete involution of the uterus within two weeks of parturition. Animals belonging to Groups A and B were fed the same diet but in a different ratio of dried grass hay tp concentrate (Group A received 6 ± 1 kg/day dried grass hay including crude protein 9%, crude fibre 35%, Ca 0.4%, P 0.23%, and 5 ± 0.5 kg/day commercially available concentrates including crude protein 16%, crude fat 6%, crude fibre 7.35%, ash 10.09%, Ca/P 1.5:1, Na 0.46%, lysine 0.85%, methionine 0.35%, omega-3 0.65%; Group B received 5 ± 0.5 kg/day hay and 2 ± 0.5 kg/day concentrates. Water was available ad libitum.

### 2.2. Data Collection and Laboratory Analysis

Body Condition Score (BCS) and body weight (BW) measurements were performed on both groups throughout the monitoring period according to previous studies [9,18]. From mares belonging to Group A, blood samples were collected by jugular venipuncture in vacutainer tubes (Terumo Corporation, Tokyo, Japan) containing ethylenediaminetetraacetic acid (EDTA) and into tubes containing clot activators (Terumo Corporation, Tokyo, Japan) on days 14 ± 2 and 7 ± 2 before foaling (T_−14_; T_−7_) and on days 7 and 14 after foaling (T_+7_; T_+14_). Blood sampling from mares belonging to Group B was performed at the beginning of the study (T_−14_). All samples were collected at the same morning time (7:00 a.m.).

Blood samples collected in EDTA tubes were centrifuged at 145× *g* for 10 min in order to obtain plasma samples. Samples from one clot activator tube were allowed to clot for two hours at room temperature, after which they were centrifuged at 1000× *g* for 20 min and the obtained sera were stored at −20 °C until analysis. The sera were analyzed in duplicate to estimate the concentration of leptin and mitochondrial uncoupling protein 1 (UCP1) using ELISA kits specific for equine species (Horse Leptin (LEP) ELISA kit MyBioSource, Inc. San Diego, CA, USA); Cat. No: MBS012684; Sensitivity 0.1 ng/mL; intra- and inter-assay coefficients of variation were at <15%; range of standard curve 0.2–20 ng/mL; Horse Uncoupling Protein 1, Mitochondrial (UCP1) ELISA Kit, (MyBioSource, Inc. San Diego, CA, USA); Cat. No: MBS066225; sensitivity 10 pg/mL; intra- and inter-assay coefficients of variation were at <15%; range of standard curve 62.5–2000 pg/mL) by means of a microtiter plate reader (EZ Read 400 ELISA, Biochrom, Cambridge, UK). All calibrators and samples were run in duplicate and samples exhibited parallel displacement to the standard curve for both ELISA analyses.

Samples from the second clot activator tube were centrifuged at 1300× *g* for 10 min, within 30 min of the collection, and the obtained sera were stored at −20 °C until analysis. The sera were analyzed to estimate the concentration of cortisol, non-esterified fatty acids (NEFAs), triglycerides, total cholesterol, high-density lipoproteins (HDLs) and low density and lipoproteins (LDLs).

Adrenocorticotrophic hormone plasma concentration, as well as the cortisol serum concentration, were measured in duplicate using a solid phase 2-site chemiluminescent immunometric assay (intra-essay coefficient of variation <5) on an Immulite 2000 analyzer (Siemens Healthcare Diagnostic, Deerfield, IL, USA) previously validated in equine species [19,20].

The serum concentration of non-esterified fatty acids (NEFAs), triglycerides, total cholesterol (TC), high density lipoproteins (HDLs) and low density and lipoproteins (LDLs) was assessed by means of an automated analyzer UV Spectrophotometer (model Slim SEAC, Florence, Italy) using commercially available kits (NEFAs, Randox, Crumlin, UK, TC, HDLs, LDLs, Byosistems, Reagents and Instruments, Barcelona, Spain). However, the values of very-low-density lipoprotein fraction (VLDLs) were estimated as one-fifth of the concentration of triglycerides [21].

### 2.3. Statistical Analysis

The data were tested for normality of distribution using the Shapiro–Wilk test. All data, expressed as mean values ± standard deviation (SD), were normally distributed (*p* > 0.05), and parametric statistical analysis was performed. Dunnett’s test was applied to evaluate the possible significant differences in considered parameters between pregnant and non-pregnant mares. One-way repeated-measures analysis of variance (ANOVA) was applied on the data obtained from mares belonging to Group A in order to assess the significant effects of time along the peripartum period on studied parameters. Tukey’s multiple comparison test was conducted when significant differences were found. Pearson’s correlation coefficients and linear regression model (y = a + bx) were computed to evaluate the possible relationship between the values of ACTH and/or cortisol and the values of leptin, UCP1 and lipid panel in the pregnant mares throughout the peripartum period.

*p*-values < 0.05 were considered statistically significant. The statistical analysis was performed using the software Prism v. 9.00 (Graphpad Software Ltd., San Diego, CA, USA, 2020).

## 3. Results

**Figure 1:**
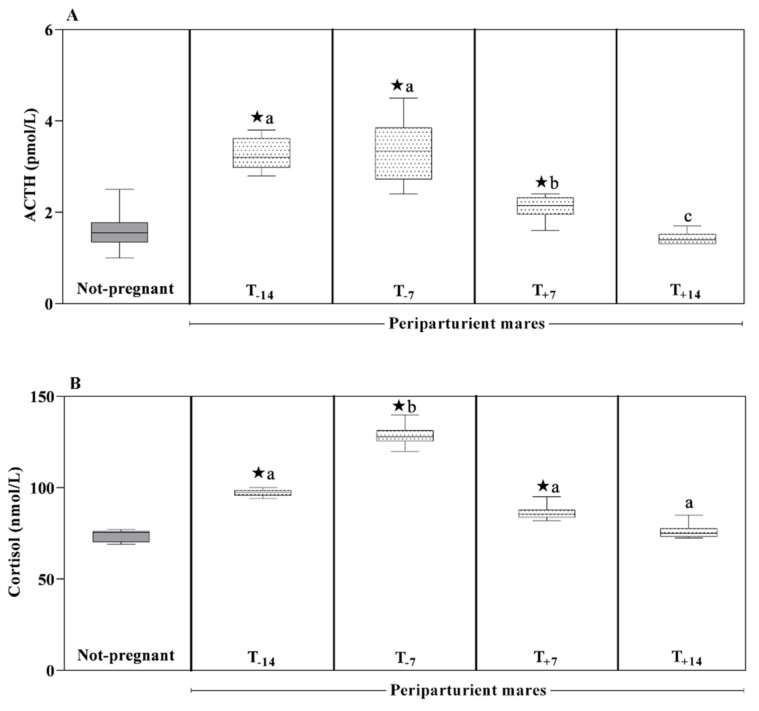
Mean values ± standard deviation (SD) of plasma adrenocorticotrophic hormone (ACTH) (**A**) and serum cortisol (**B**) obtained in pregnant (Group A) and not-pregnant mares (Group B) with relative statistical significances. The symbol “star” (★) reflects difference significant differences between groups vs. non-pregnant mares (*p* < 0.0001); Different alphabetic letters show significant differences within peripartum period (*p* < 0.0001).

No statistically significant changes in BCS were observed between Groups A and B and in pregnant mares over time. The BW values statistically differed (*p* < 0.05) between groups during the prepartum period only. Prepartum and postpartum BW values recorded from Group A statistically changed (*p* < 0.05), whereas no difference was found in BW over time within the prepartum and postpartum intervals (*p* > 0.05). A significant effect of peripartum time on plasma ACTH and serum cortisol values measured in mares of Group A was found (*p* < 0.0001). In particular, Group A showed higher plasma ACTH values at T-7 compared to T_+7_ and T_+14_ and lower values at T_+14_ compared to T_+7_ and T_−14_. Serum cortisol levels measured in mares from Group A were statistically significantly higher at T_+7_ compared to the other peripartum time points (Figure 1A,B).

**Figure 2:**
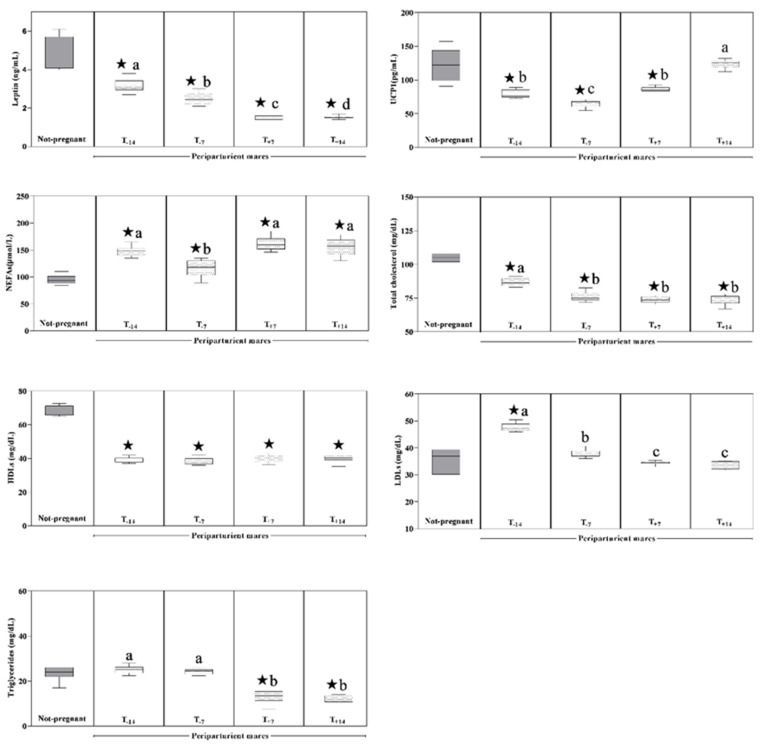
Mean values ± standard deviation (SD) of serum leptin, mitochondrial uncoupling protein 1 (UCP1), non-esterified fatty acids (NEFAs), total cholesterol, high-density lipoproteins (HDLs), low-density lipoproteins (LDLs) and triglycerides obtained in periparturient mares (Group A) and non-pregnant group (Group B) with the relative statistical significances. The symbol “star” (★) reflects difference significant differences between groups, vs. non-pregnant mares (*p* < 0.0001); different alphabetic letters show significant differences within peripartum period (*p* < 0.0001).

As displayed in Figure 2, a decreasing trend of leptin concentration was found in mares belonging to Group A starting from the prepartum period until the postpartum period, whereas serum UCP1 showed decreased values during the prepartum period following by a rise until the second week after parturition (*p* > 0.0001). The serum triglycerides, TC, VLDLs and LDLs showed lower values at the postpartum data points compared to the prepartum data points (*p* < 0.0001). Serum NEFAs values were higher at T_−14_ than T_−7_ and at T_+7_ and T_+14_ compared to T_−7_ (*p* < 0.0001). The HDLs levels remained statistically unchanged (*p* > 0.05), showing a constant trend throughout the monitoring period (Figure 2). A significant effect of pregnancy (*p* < 0.0001) was found on all studied parameters (Figure 1 and Figure 2). In particular, Group A showed statistically significant higher plasma ACTH and serum cortisol levels at the considered prepartum time points (T_−14_, T_−7_) and one week after foaling (T_+7_) than the values measured in non-pregnant mares. Lower serum UCP1 values were found in Group A than Group B at all sampling times except at T_+14_, when UCP1 showed increased values up to the levels measured in the control group. Lower serum values of leptin, total cholesterol and HDLs levels and higher serum NEFAs values were found in Group A than in the non-pregnant group during the survey. After parturition, Group A showed lower serum triglycerides and VLDLs levels than control, whereas LDLs showed higher serum levels in Group A than Group B two weeks before parturition.

**Figure 3:**
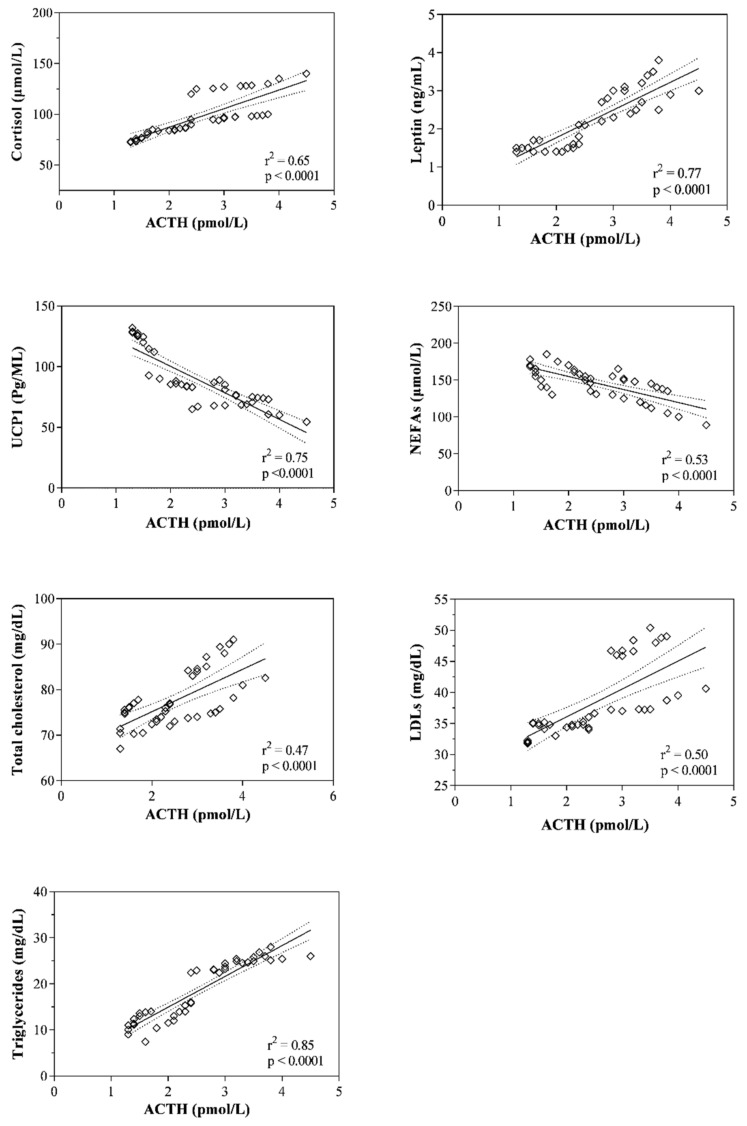
Linear regression values between the plasma adrenocorticotrophic hormone (ACTH) concentration and the levels of the serum parameters resulted statistically correlated in pregnant mares (Group A) throughout the considered peripartum period (i.e., cortisol, leptin, mitochondrial uncoupling protein 1, UCP1; non-esterified fatty acids, NEFAs; total cholesterol, low density and lipoproteins, LDLs; triglycerides; very-low-density lipoprotein fraction, VLDLs).

**Figure 4:**
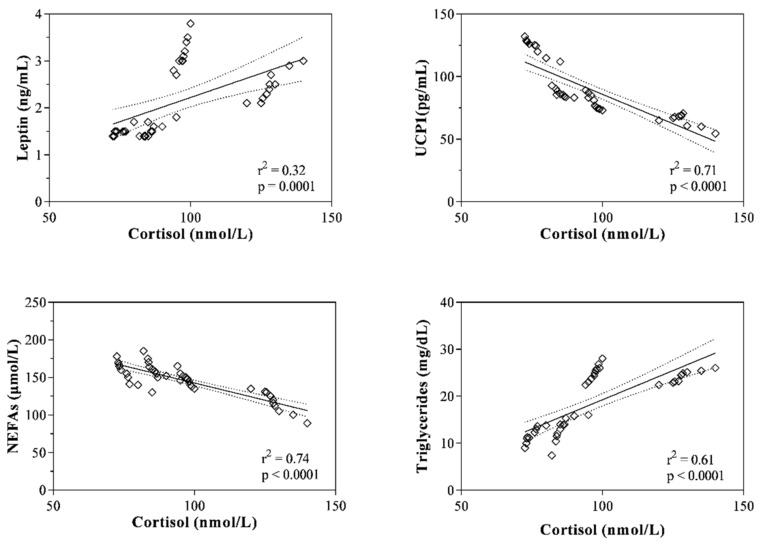
Linear regression values between the serum cortisol concentration and the levels of serum parameters were statistically correlated in pregnant mares (Group A) throughout the considered peripartum period (i.e., leptin; mitochondrial uncoupling protein 1, UCP1; non-esterified fatty acids, NEFAs; triglycerides; very low-density lipoprotein fraction).

The plasma ACTH and serum cortisol levels measured in mares belonging to Group A showed a significant positive correlation with the serum values of leptin, LDLs, triglycerides and VLDLs, whereas plasma ACTH and serum cortisol resulted negatively correlated with serum UCP1 and NEFAs values (Table 1). The statistically significant correlations found were confirmed by the linear regression model results (Figure 3 and Figure 4).

## 4. Discussion

Knowledge of functional adaptation of mares throughout a stress condition as the peripartum period is crucial for the monitoring of their health status and for the prompt diagnosis of pathologic conditions that could jeopardize both the mare’s and the fetus’s life. The normal function of the HPA axis is important for the maintenance of homeostasis during stressful conditions [8], whereas the constant release of stress hormones by repeated stress is a characteristic sign of adverse health outcomes [22]. The studies of situations associated with more prolonged stress have shown inceases in plasma cholesterol, triglycerides and lipoproteins, and as chronic stress may induce changes in glucocorticoids, a connection between the HPA axis and adipose tissue has been suggested [3,4,23]. The findings obtained in the current study revealed that ACTH and cortisol followed the same trend throughout the monitoring period, as underlined by the significant positive correlation found between these hormones throughout the study period. Despite the dynamic changes found herein, the ACTH and cortisol values were within reference intervals established for equine species [24].

The increase in ACTH and cortisol in pregnant compared to non-pregnant animals is not surprising; indeed, during pregnancy, placenta and fetal membranes produce CRH, which stimulates maternal ACTH secretion, which in turn stimulates the biosynthesis of cortisol [24]. Moreover, according to findings obtained in the human species [25], and justifying the higher values of ACTH and cortisol found in pregnant mares than non-pregnant mares, it is well known that during pregnancy, the maternal adrenal glands become hypertrophic with increased ACTH secretion and cortisol production. Moreover, ACTH and cortisol measured in mares belonging to Group A showed decreased levels after foaling. The decline of the ACTH and cortisol levels after parturition could point out a diminished stress response of the postpartum mares, as a matter of fact, a reduction in stress reaction is known to occur in animals after parturition [26,27]. Furthermore, the postpartum hyperprolactinemia occurring in lactating mammals seems to lead to the HPA axis attenuation to a stressor [27,28]. Humans surveys hypothesized that the HPA axis attenuation during postpartum is an adaptive response occurring in dams to optimize maternal care by reducing anxiety [26,29].

It has been suggested that during lactation, the energy signaling leads to the mobilization of fatty acids from adipose tissue and feed intake in order to allow mares to maintain good body condition and an adequate milk supply [9,30]. In this regard, the functions of the HPA axis and the systemic sympathetic/adrenomedullary system are modulated by leptin through its receptors located in the hypothalamus [31,32]. Intensified HPA axis activity leads to fat cell growth, insulin resistance, increased food intake, decreased thermogenesis and energy expenditure [33,34]. Overall, this evidence seems to justify the relationship between the levels of ACTH and/or cortisol and the trend of leptin, UCP1 and lipid indices found herein. Specifically, both HPA axis hormones were negatively correlated with UCP1 and NEFAs values. These findings seem to confirm the conclusions of previous studies which suggest that increased activity of the HPA axis leads to decreased thermogenesis and energy expenditure [33,34]. Moreover, ACTH was positively correlated with the serum values of leptin, total cholesterol, LDLs, triglycerides and VLDLs, whereas serum cortisol levels showed a positive correlation with triglycerides and VLDLs in periparturient mares. The findings gathered by correlation analysis seem to emphasize the hypothesis of a relationship between the HPA axis and lipid metabolism in mares during the postpartum period. According to this hypothesis, previous studies showed a link between the HPA axis and adipose tissue, where glucocorticoids and ACTH can stimulate leptin secretion by fat cells [11,23]. To date, the mechanisms by which leptin modulates the HPA axis functions at the hypothalamus level are not fully identified. It has been suggested that leptin inhibits the stress-responsive secretion of hypothalamic corticotropin-releasing hormone (CRH) in mice [34,35,36], whereas it has been shown that high amounts of glucocorticoids lead to increased leptin expression in vitro [37] and its secretion in vivo [38]. Moreover, study of situations associated with prolonged stress with the enrolment of ACTH and cortisol secretion showed rises in lipid indices, suggesting lipolysis enhancement [39].

Several factors such as body fat mass, age, gender, nutritional programs and environmental conditions could influence the serum concentration of leptin and of the other lipid parameters investigated herein [40,41]. However, in the present study, the mares were selected with similar BCS, age and nutritional diet to minimize differences caused by different metabolic conditions and to allow the analysis of serum parameters levels depending on the reproductive state of the mares. Contrary to the results obtained in studies carried out on humans, rats and sheep highlighting a pregnancy-associated increase in leptin concentrations [42,43], the findings obtained herein showed lower leptin values in pregnant mares than in non-pregnant mares throughout the study period and a decreasing trend in Group A following parturition. Leptin, synthetized in adipose tissue, is released into the bloodstream proportionally to the overall degree of adiposity; the leptin release provides the central nervous system with information on metabolic state and energy balance of the organism [40,41,44]. In accordance with previous surveys carried out on periparturient mares [9,45,46,47], a decrease in leptin concentration following parturition was observed herein in investigated mares, as this tissue can also produce leptin [48,49]. The concentration decrease in this hormone could be due to the loss of placenta, a tissue likely known to produce leptin [48,49], and may represent a valuable tool against negative energy balance in mares by encouraging feed intake during early lactation [45].

It is known that serum leptin levels influence the UCP1 expression [12,50], accordingly, the results found herein showed that serum values of UCP1 and leptin followed the same trend during prepartum times, whereas, after foaling, UCP1 showed increased values, while leptin continued to decrease throughout the postpartum time. The dynamic changes showed by UCP1 values in pregnant mares during the peripartum period and in comparison to non-pregnant mares could be related to the hormonal pathway controlling the peripartum period to ensure energy homeostasis.

During pregnancy, the expression of UCP1 is regulated by estrogens, which modulate both the thermogenic potential of brown adipose tissue and the distribution pattern of adipose tissue by an increase in sympathetic outflow on brown adipose tissue, leading to the increase in thermogenic mechanisms such as UCP1 expression [51,52]. Noteably, according to the UCP1 trend observed herein, it seems that the effects of estrogen are abolished during pregnancy. This hypothesis agrees with previous results obtained in pregnant rats displaying a thermogenesis and brown adipose tissue function reduction [53]. Therefore, it could be hypothesized that, in order to guarantee gestational hyperphagia and adiposity, pregnancy induces a state of resistance to the thermogenic action of estrogens. This could represent an adaptation mechanism put in place by the organism to cope with the metabolic demands of fetus development and beginning of lactation [54]. The increase in serum UCP1 levels observed during postpartum seems to suggest that estrogens recuperate their stimulating function on UCP1 expression in mares after parturition [54]. Moreover, the higher NEFAs values measured in Group A than Group B as well as the increasing trend observed in Group A throughout the postpartum time points could be related to the milk-fat synthesis by mammary tissue. Indeed, although the fat content in mare milk is relatively low (1.3–2.1%) [55], lactation would still require an increased supply of fatty acids for lipid synthesis. Therefore, the rise in serum NEFAs levels after foaling might be related to increased lipolysis of VLDL triglyceride and/or to the mobilization of NEFAs from adipose tissue to meet the increased demands due to milk production. According to previous studies [2,56], serum triglycerides and VLDLs showed lower levels in Group A after parturition than the prepartum times. The trend found for these lipids is probably linked to lipid transfer through both the placenta for fetal energy supplies and the mammary gland for milk yield [50,57]. Even though they are within the physiological range [58], lower total cholesterol and HDLs values and higher LDLs levels were found in Group A compared to non-pregnant mares throughout the monitoring period. Total cholesterol and LDLs showed a decreasing trend from the prepartum until the postpartum time in Group A; moreover, lower total cholesterol concentration was found in pregnant mares compared to the non-pregnant group. The total cholesterol decrease after foaling might be due to lactation [59,60], whereas the difference in the concentration of this lipid found between groups might be related to the uptake of cholesterol by organs such as ovaries and placenta for steroid hormone synthesis during pregnancy [59,60]. It is not surprising that the levels of total cholesterol and of LDLs paralleled each other as LDLs represent the main pathway by which cells of the ovaries, placenta and mammary gland acquire cholesterol to synthetize steroids and to produce milk, respectively [60].

## 5. Conclusions

An adequate animal adaptive response to the increased metabolic demands and energetic needs of ovary, placenta and mammary glands, is crucial for the periparturient mare to maintain a good body condition score avoiding improper energy expenditure, lipolysis and fat utilization. The results found in the current study highlight the dynamic modulation of serum leptin, UCP1, lipids and lipoproteins levels as a response to hormonal and metabolic adaptations occurring during late pregnancy and early postpartum period in mares. The differences in ACTH and cortisol associated with the dynamic change of leptin, UCP1 and lipid parameters herein investigated might indicate an interaction of HPA axis with lipid metabolism and mobilization in mares during the peripartum period in order to maintain energy homeostasis. The current survey serves as a starting point for future study of the cause-and-effect nature of these interactions in equine species during peculiar life periods as pregnancy and lactation.

## Figures and Tables

**Table 1:** Coefficients of correlation among the values of plasma ACTH and/or serum cortisol and the serum concentration of leptin, mitochondrial uncoupling protein 1 (UCP1), non-esterified fatty acids (NEFAs), total cholesterol, high-density lipoproteins (HDLs), low-density lipoproteins (LDLs) and triglycerides calculated for mares of Group A during the peripartum period. *P*-values < 0.05 were considered statistically significant.

	Cortisol (nmol/L)	Leptin (ng/mL)	UCP1 (pg/mL)	NEFAs (µmol/L)	Total Cholesterol (mg/dL)	HDLs (mg/dL)	LDLs (mg/dL)	Triglycerides (mg/dL)
**ACTH** (pmol/L)	r = 0.81 *p* < 0.0001	r = 0.88 *p* < 0.0001	r = −0.87 *p* < 0.0001	r = −0.73 *p* < 0.0001	r = 0.68 *p* < 0.0001	r = 0.07 *p* = 0.67	r = 0.71 *p* < 0.0001	r = 0.92 *p* < 0.0001
**Cortisol** (nmol/L)		r = 0.57 *p* = 0.0001	r = −0.86 *p* < 0.0001	r= −0.86 *p* < 0.0001	r= 0.22 *p* = 0.19	r = −0.14 *p* = 0.37	r = 0.28 *p* = 0.08	r = 0.71 *p* < 0.0001

## Data Availability

The data presented in this study are available on request from the corresponding author.
